# Causal Associations Between Smoking, Brain Structural Alterations and Psychiatric Disorders: Evidence From a Mediation Analysis

**DOI:** 10.1111/adb.70102

**Published:** 2025-11-25

**Authors:** Yang Chen, Xiaoying Ma, Yubing Yin, Yulu Wu, Yunqi Huang, Yiguo Tang, Siyi Liu, Qianshu Ma, Menghan Wei, Mengting Zhang, Shiwan Tao, Min Xie, Renhao Deng, Mingli Li, Qiang Wang

**Affiliations:** ^1^ Mental Health Center West China Hospital of Sichuan University Chengdu Sichuan China; ^2^ Sichuan Clinical Medical Research Center for Mental Disorders Chengdu Sichuan China

**Keywords:** imaging‐derived phenotypes (IDPs), Mendelian randomization, psychiatric disorders, SH2B2 gene, smoking behaviour

## Abstract

Both epidemiological and Mendelian randomization (MR) studies have confirmed the association between smoking and psychiatric disorders, yet the underlying mechanism remains poorly understood. To address this gap, this study aimed to evaluate causal relationships between smoking, brain structural alterations, and psychiatric disorders and to identify genetic and neuroimaging mediators. We analysed summary data from the genome‐wide association study (GWAS) and multimodal neuroimaging data, using linkage disequilibrium score regression (LDSC) to quantify genetic correlations and two‐sample bidirectional MR to assess causality between smoking and three psychiatric disorders: schizophrenia (SCZ), major depressive disorder (MDD) and bipolar disorder (BD). Additionally, we conducted mediation analysis to identify brain structural mediators and colocalization and pathway analyses to elucidate shared genetic architecture. LDSC analysis revealed significant genetic correlations between smoking and MDD (*r*_*g*_ = 0.41), SCZ (*r*_*g*_ = 0.16) and BD (*r*_*g*_ = 0.15). Consistently, bidirectional MR confirmed a bidirectional causal relationship between smoking and SCZ/MDD and a unidirectional causal effect of smoking on BD. Mediation analysis further revealed that microstructural disorganization in the left uncinate fasciculus mediated 19.6% (95% CI: 3.36%‐35.8%) of the effect of smoking on MDD risk. Moreover, colocalization analysis implicated SH2B2 as a pleiotropic locus linking UF orientation dispersion (OD) to prefrontal–amygdala gene expression, suggesting that smoking may exacerbate corticolimbic dysfunction by inducing SH2B2 hypermethylation. Taken together, our findings establish smoking as a causal risk factor for SCZ, MDD and BD, with corticolimbic white matter degeneration serving as a key mediator, and they highlight the SH2B2‐TrkB axis as a mechanistic conduit for genetic and environmental risk, pointing to therapeutic targets to disrupt smoking related psychiatric disorders.

## Graphical abstract

This study establishes smoking as a causal risk factor for schizophrenia, major depression and bipolar disorder using genetic evidence. We identified microstructural disorganization in the left uncinate fasciculus, a key corticolimbic white matter tract, as a significant mediator of this risk, explaining 19.6% of the effect on depression. Genetic analyses further implicated the SH2B2 gene, suggesting a mechanism whereby smoking may induce epigenetic changes that disrupt prefrontal‐amygdala circuitry. These findings highlight white matter degeneration and the SH2B2‐TrkB axis as pivotal contributors to smoking‐related psychiatric pathology, revealing new potential therapeutic targets.

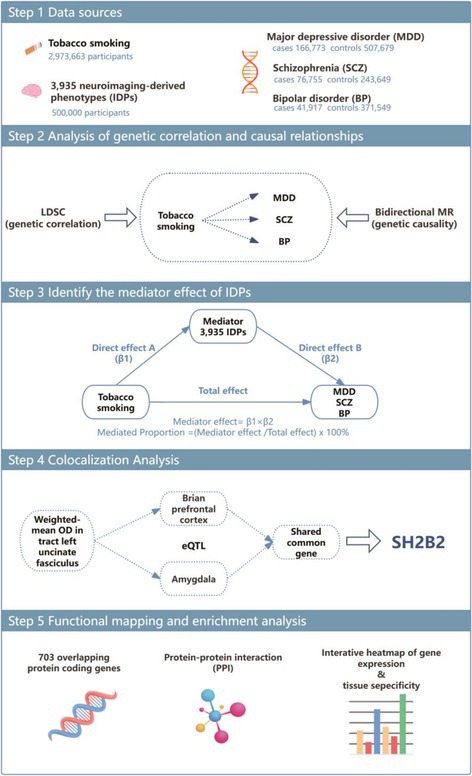

## 1 Introduction

Psychiatric disorders, including major depressive disorder (MDD), bipolar disorder (BD) and schizophrenia (SCZ), are a significant, highly prevalent public health challenge affecting approximately one billion of the global population. Both epidemiological and socioeconomic studies around the world have demonstrated that psychiatric disorders are a leading cause of global disease burden in terms of disabilities, premature mortality and economic costs [1]. Therefore, it is crucial to identify modifiable risk factors of psychiatric disorders to inform future cost‐effective public health approaches to prevention.

Smoking is a well‐established risk factor for psychiatric disorders, with epidemiological studies indicating a 2–5 times higher prevalence of smoking in individuals with psychiatric disorders, particularly BD and SCZ, than that of the general population [2, 3, 4]. Additionally, smoking is significantly associated with reduced life expectancy among individuals with psychiatric disorders, with a study showing a decrease in life expectancy by 7.3 years in female smokers and 5 years in male smokers diagnosed with BD or SCZ [5]. The observed significant link between smoking and psychiatric disorders was further corroborated by more robust evidence based on Mendelian randomization (MR) analyses [6, 7, 8]. By utilizing genetic variation to infer causal relationships between the exposures and outcomes, MR addresses the potential confounding and reverse causation associated with traditional observational studies [9]. A recent literature review across 63 MR studies confirmed a bidirectional, increasing association between smoking and psychiatric disorders, suggesting a shared genetic susceptibility underlying their comorbidity [10].

Although both observational and MR studies have shown positive associations between smoking and psychiatric disorders, the precise genetic architecture and specific susceptibility genes remain incompletely understood. Recent multimodal neuroimaging investigations reveal that smoking and psychiatric disorders share overlapping neuropathological features, leading to similar changes in brain structures and functions [11, 12]. For instance, cortical thinning of the prefrontal cortex (PFC) and reduced integrity of white matter tracts, such as the chiasmic tracts, have been documented in both smokers and patients with SCZ [13], BD [14] and MDD [15]. However, the complex relationships between genetic predisposition, brain structural alterations and their combined influence on smoking and psychiatric disorders remain poorly studied.

To fill in the research gap, we conducted a two‐sample MR study to systematically explore the potential causal connections between smoking, brain structural changes and the risk of three major psychiatric disorders: SCZ, BD and MDD. Specifically, we performed the bidirectional MR analysis to investigate the bidirectional causality between smoking and psychiatric disorders. Additionally, we performed the mediation analysis to explore and quantify the mediating effects and genetic mechanisms of brain structural alterations in the causal association between smoking and psychiatric disorders. This study would offer comprehensive insights into how smoking may influence psychiatric disorders through neurobiological mechanisms. These findings can guide future development of targeted and effective prevention and treatment approaches for psychiatric disorders, especially in individuals with co‐occurring smoking.

## 2 Methods

### 2.1 Genome‐Wide Association Study (GWAS) Data Sources

#### 2.1.1 Smoking

GWAS data on smoking behaviours were derived from GSCAN, FinnGen (Freeze 10; https://r11.finngen.fi/) and the UK Biobank. The dataset was obtained from the GWAS Catalogue ‘GCST90267302’. GWAS datasets of independent European ancestry were selected to prevent the overlap of samples [16]. The GSCAN dataset included 2 669 029 Europeans, who were categorized as ever‐smokers and never‐smokers based on the binary variable ‘smoking initiation’ (SmkInit) [17]. The FinnGen and UK Biobank datasets consisted of 20 885 and 283 749 individuals, respectively. The METAL software was employed to perform a fixed‐effect inverse‐variance weighted (IVW) meta‐analysis from three cohorts.

#### 2.1.2 Psychiatric Disorders

We obtained GWAS summary statistics for the three psychiatric disorders from the Psychiatric Genomics Consortium (PGC) and iPSYCH. To control population‐based bias, we selected genetic data from the European population, ensuring consistency with the exposure data. The sample sizes for each disorder were as follows: SCZ (76 755 cases and 243 649 controls) [18], BP (41 917 cases and 371 549 controls) [19] and MDD (166 773 cases and 507 679 controls) [20]. Quality control procedures were applied to ensure robust and reliable results.

#### 2.1.3 Neuroimaging‐Derived Phenotypes (IDPs)

GWAS summary statistics for IDPs were obtained from the UK Biobank, encompassing genomic and phenotypic data from over 500 000 individuals [21]. The UK Biobank neuroimaging protocol implemented six clinically validated MRI sequences: T1‐weighted structural imaging, T2‐weighted fluid‐attenuated inversion recovery (T2‐FLAIR), diffusion MRI (dMRI), resting‐state functional MRI (rfMRI), task‐fMRI (t‐fMRI) and susceptibility‐weighted imaging (SWI). Through standardized preprocessing pipelines involving FSL, Freesurfer and DTIPrep tools, 3935 quantitative IDPs were algorithmically derived from multimodal MRI data, spanning structural connectivity, functional activation patterns and microstructural indices (Supporting Information: Table 2). Prior GWAS consortia have analysed these IDPs in European‐ancestry subsamples (*N* = 33 224), identifying loci meeting genome‐wide significance (*p <* 5 *×* 10^*−*8^).

#### 2.1.4 GTEx eQTL Data

We obtained data on the brain frontal cortex and amygdala expression quantitative trait loci (eQTL) from GTEx v8 [22], including 15 201 RNA‐sequencing samples from 49 tissues of 838 postmortem donors.

### 2.2 Linkage Disequilibrium Score (LDSC) Regression Analysis

We estimated the genetic correlations (*r*_*g*_) between smoking and three major psychiatric disorders (BP, MDD and SCZ) using LDSC. LDSC quantifies inflation in test statistics and distinguishes between inflation caused by polygenic signal and population stratification. It ensures the unbiased estimation of genetic correlations from GWAS summary data, even in the presence of sample overlap.

### 2.3 Instrumental Variables (IV) Selection

We processed the GWAS summary statistics following a series of steps. We excluded SNPs located within the major histocompatibility complex (MHC) region, SNPs with a minor MAF < 0.01 and palindromic SNPs (i.e., those with A/T or G/C alleles) when their MAF was nearly 0.5. Genetic IVs for MR were selected based on the three key criteria: (1) relevance, the IV was significantly associated with the exposure, with genome‐wide significance at *p <* 5 *×* 10^*−*8^; (2) independence, there were no other confounders and no linkage disequilibrium (using an *r* ^2^ threshold > 0.001 within a 1000, kb window); and (3) exclusion restriction, the IV should only affect the outcome through exposure, without weak instrument bias (*F*‐statistic > 10). Finally, we conducted harmonization of the effect alleles and adjustment of the outcome data to ensure that the exposure and outcome datasets were consistent.

### 2.4 MR Analysis

#### 2.4.1 Primary Analysis

We conducted a bidirectional two‐sample *MR* analysis to estimate each of the following total causal effects. We used a variety of methods to estimate the *MR* effects and improve the robustness. The primary technique was *IVW*, complemented by the *MR*‐Egger and weighted median approaches. These methods differ in their assumptions about IV validity. In particular, the *IVW* method assumes that all *SNP*s function as valid IVs, thus providing accurate estimates. In contrast, the *MR*‐Egger method evaluates directional pleiotropy by using its intercept to estimate the average pleiotropic effect of the IVs. Furthermore, the weighted median approach has higher precision and lower standard deviation compared to *MR*‐Egger.

#### 2.4.2 Mediation MR Analysis

In addition, a two‐step MR approach was used to explore the potential mediating effect of IDPs in the causal pathway between smoking and three major psychiatric disorders. The total causal effect of smoking on psychiatric disorders was partitioned into two pathways:(1) a direct effect from smoking to psychiatric disorders and (2) an indirect effect mediated by IDPs. The delta method of mediation testing was used to quantitatively assess the strength of mediation. Furthermore, we applied the Sobel test for standard‐error adjustment of the mediated effect and for two‐sided significance testing. We calculated the proportion of the indirect effect, relative to the total effect and derived the associated 95% confidence intervals for statistical inference. To mitigate bias arising from potential sample overlap between the exposure and outcome GWAS, we additionally applied the MRlap framework to obtain overlap‐adjusted effect estimates.

#### 2.4.3 Pleiotropy and Heterogeneity Analysis

To validate our causal findings, we conducted comprehensive analyses of pleiotropy and heterogeneity. Specifically, we employed the MR‐PRESSO method to remove outlier SNPs and reanalyze the dataset. We used MR‐Egger regression to evaluate horizontal pleiotropy, with an intercept yielding *p >* 0.05 indicating no directional pleiotropy. We assessed IV heterogeneity using both MR‐Egger and IVW approaches, with a Cochran's Q statistic yielding *p >* 0.05 suggesting nonsignificant heterogeneity. Additionally, we performed sensitivity analyses using a leave‐one‐out procedure, which excluded individual SNPs sequentially to determine their influence on the exposure–outcome associations. All statistical analyses were executed using R software (version 4.4.1) with the TwoSampleMR and MR‐PRESSO packages. A two‐sided *p* < 0.05 indicates statistical significance.

### 2.5 Colocalization Analysis

Bayesian colocalization analysis was employed to assess the relationship between UF orientation dispersion (OD) and eQTL in the PFC and amygdala. We set the a priori probability of a random variant being associated with GWAS data to 5 *×* 10^*−*8^ and searched for each gene in the 1 Mb range of sentinel SNPs with the xQTLanalyzegetTraits function. We derived the posterior probability (PPH4) and HyPr‐posterior for each gene and considered both values of 0.9 and above as colocalization. Bayesian colocalization analyses were conducted using the R package xQTLbiobinks [23].

### 2.6 Functional Mapping and Enrichment Analysis

FUMA is a GWAS post‐annotation programme that was used to develop a range of SNP functional annotation methods. We used FUMA to annotate genes obtained from colocalization. We employed protein–protein interaction (PPI) analyses using STRING version 12.0 for protein‐coding genes that overlap between the GWAS datasets for smoking, depression and the weighted‐mean OD in the tract left uncinate fasciculus.

## 3 Result

### 3.1 LDSC Regression Analysis

LDSC regression analysis showed that the genetic correlations between smoking and MDD, BP and SCZ were 0.4102 (*p* = 8.737 *×* 10^*−*88^), 0.1544 (*p* = 5.5233 *×* 10^*−*14^) and 0.163 (*p* = 7.3113 *×* 10^*−*18^), respectively. These results suggested a significant common genetic basis for smoking and the three psychiatric disorders. The LDSC intercepts for MDD (1.0289, SE = 0.0105), BP (1.0174, SE = 0.009), SCZ (0.012, SE = 0.0189) and smoking (0.7927, SE = 0.0098) suggested that the inflation observed in the test statistic was primarily driven by polygenic structure rather than confounding factors such as population stratification (Table 1).

**TABLE 1:** Genetic correlation between smoking and three severe mental disorders.

Phenotype	h^2^	r_g_	*p*‐Value	SE
Smoking	0.01	—	—	—
SCZ	0.37	0.163	7.3113e‐18	0.0189
BP	0.07	0.154	5.5233e‐14	0.0205
MDD	0.06	0.410	8.737e‐88	0.0207

Abbreviations: BP, bipolar disorder; MDD, major depressive disorder, SCZ, schizophrenia.

### 3.2 MR Analysis

#### 3.2.1 Association of Genetically Predicted Smoking With Psychiatric Disorders

Unidirectional MR analyses showed that genetic predisposition to smoking initiation significantly elevated risks for MDD (IVW OR = 1.718, 95%CI = 1.480–1.995, *p* = 1.144 × 10^−12^), BD (IVW OR = 3.960, 95%CI = 2.727–5.751, *p* = 4.774 × 10^−13^) and SCZ (IVW OR = 3.744, 95%CI = 2.712–5.169, *p* = 1.022 × 10^−15^).

**FIGURE 1:**
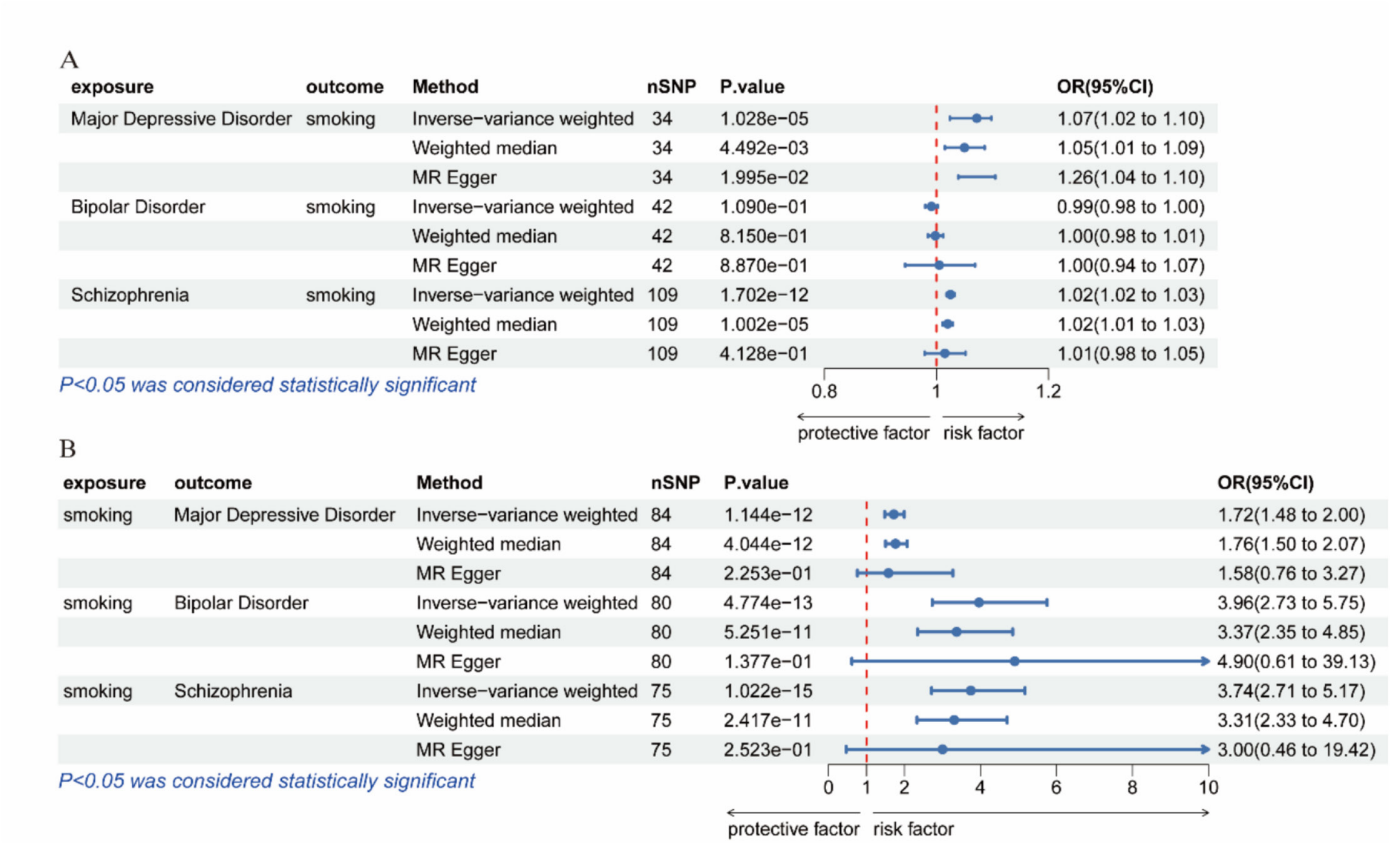
The Forest plot of MR analysis represents the causal relationship between smoking and three major psychiatric disorders. (A) Causal effects of smoking on psychiatric disorders. (B) Causal effects of psychiatric disorders on smoking. CI, confidence interval; OR, odds ratio; P, *p*‐value.

Reverse MR analyses showed that both genetic susceptibility to MDD (IVW OR = 1.072, 95%CI = 1.024–1.098, *p* = 1.028 × 10^−5^) and SCZ (IVW OR = 1.025, 95%CI = 1.018–1.032, *p* = 1.702 × 10^−12^) significantly increased the risk of smoking initiation. However, genetic susceptibility to BD did not show any significant effect on smoking (Table 2, Figure 1).

**TABLE 2:** Mediation effect of weighted‐mean OD in tract left uncinate fasciculus between smoking and major depressive disorder.

Mediator	Outcome	Total effect *β* (95% CI)	Direct effect A *β* (95%CI)	Direct effect B *β* (95%CI)	Mediator effect(95%CI)	Mediated proportion	*p*
Weighted‐mean OD in tract left uncinate fasciculus	Major depressive disorder	0.541 (0.392,0.691) *p* < 0.005	0.268 (0.097,0.438) *p* < 0.005	0.396 (0.207,0.586) *p* < 0.005	0.106 (0.0182, 0.194)	19.6%	0.018

Robustness assessments confirmed the methodological validity. MR‐Egger regression detected no directional pleiotropy (intercept *p*‐values > 0.05 across all models). Funnel plots demonstrated balanced effect size distributions. Iterative leave‐one‐out sensitivity analyses revealed stable causal estimates, with the sequential exclusion of individual SNPs producing consistent magnitude and directionality of effects (Supporting Information: Table 1 and Supporting Information: Figures 1–2).

#### 3.2.2 Association of Genetically Predicted Smoking With IDPs and IDPs With Psychiatric Disorders

**FIGURE 2:**
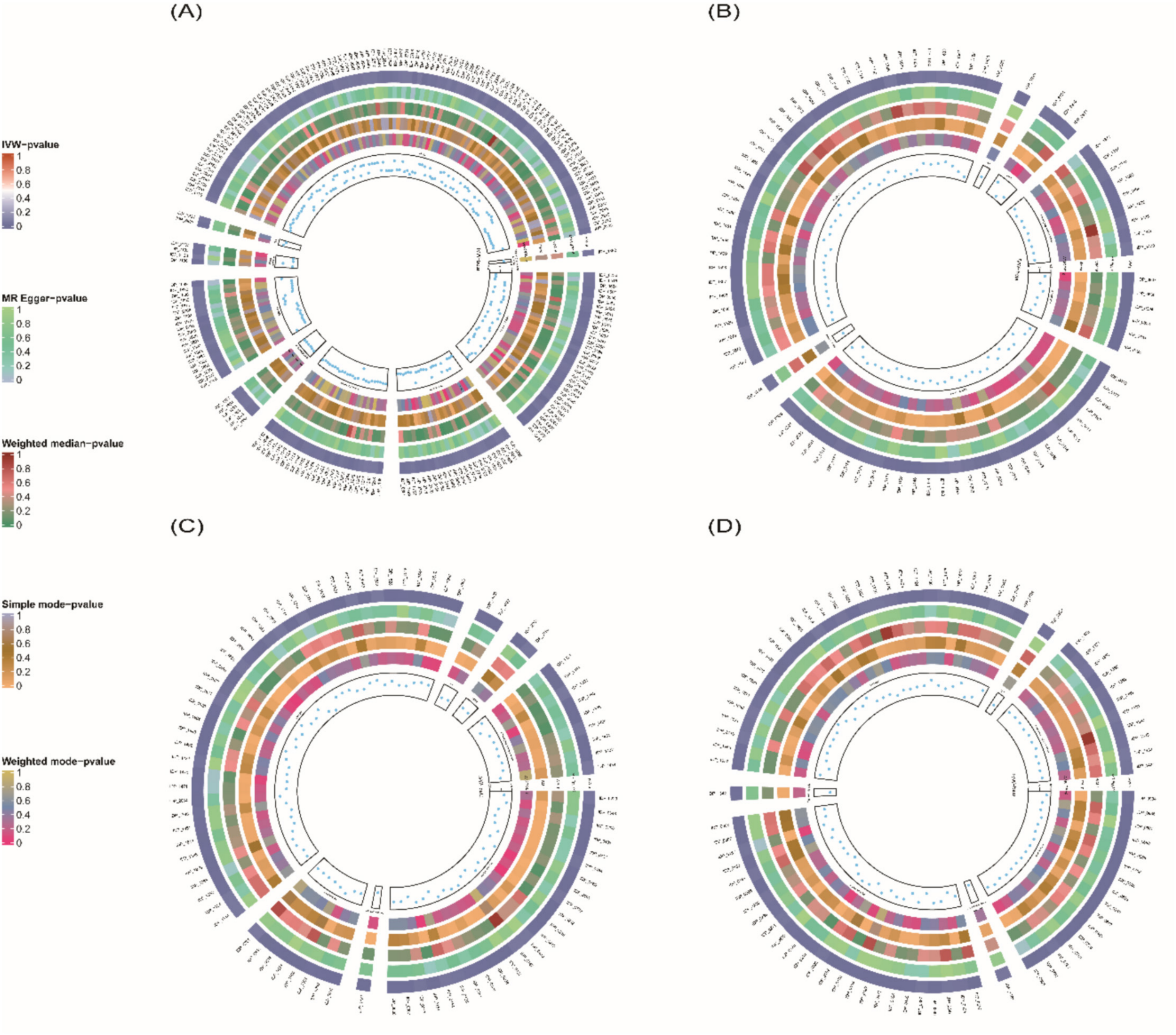
Comprehensive visualization of significant causal relationships identified through Mendelian randomization analysis. (A) Circular plot showing significant positive associations (IVW *p* < 0.05) from smoking exposure to 3935 brain imaging‐derived phenotypes (IDPs). (B) Circular plot depicting significant positive associations (IVW *p* < 0.05) from 3935 brain IDPs to MDD. This represents the causal influence of brain imaging phenotypes on MDD risk. (C) Circular plot depicting significant positive associations (IVW *p* < 0.05) from 3935 brain IDPs to Schizophrenia (SCZ). This represents the causal influence of brain imaging phenotypes on SCZ risk. (D) Circular plot depicting significant positive associations (IVW *p* < 0.05) from 3935 brain IDPs to bipolar disorder (BP). This represents the causal influence of brain imaging phenotypes on BP risk.

**FIGURE 3:**
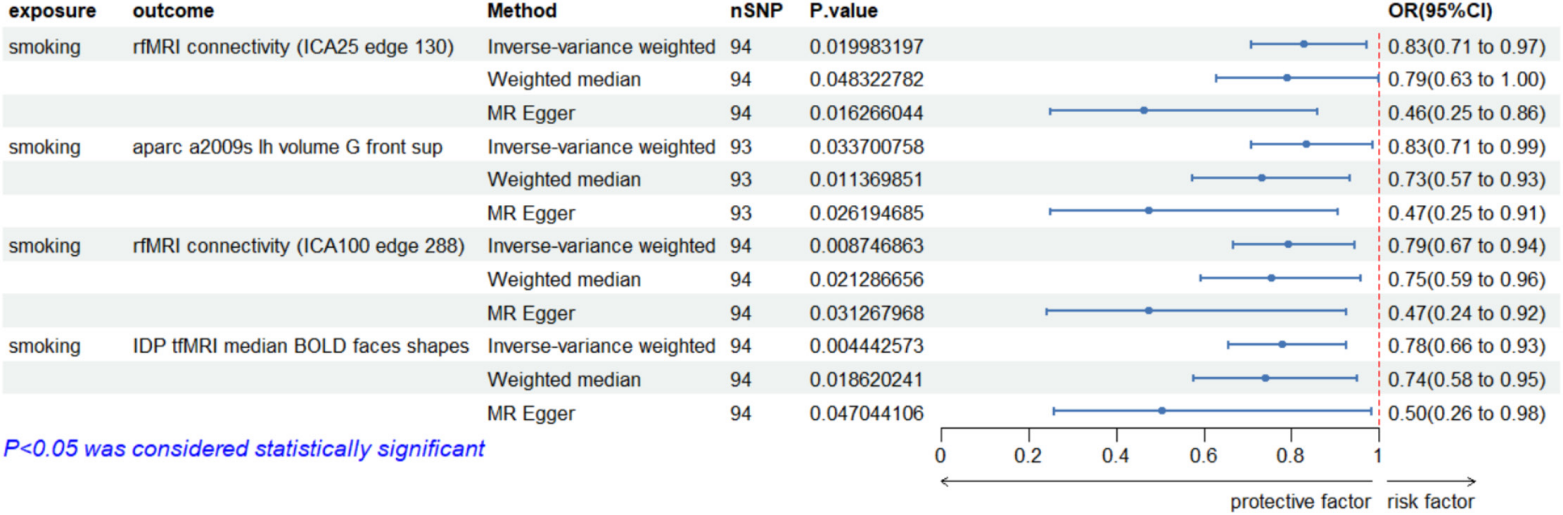
Forest plot of causal relationships between smoking and brain imaging‐derived phenotypes (IDPs) identified through Mendelian randomization analysis. This figure presents results from Mendelian randomization analyses examining the causal effect of smoking on brain imaging‐derived phenotypes. Only outcomes that showed statistical significance across all three MR methods (IVW, weighted median and MR‐Egger) are displayed.

The two‐sample MR analysis identified that smoking was causally associated with 216 brain IDPs. Additionally, 84, 85 and 85 brain lDPs were causally associated with BP, MDD and SCZ, respectively (*p* < 0.05 in IVW analyses) (Figure 2). Further analysis showed that smoking was causally associated with 4 brain lDPs (*p* < 0.05 in MR‐Egger, IVW and weighted median) (Figure 3).

Multiple sensitivity analyses were conducted using diverse methodological approaches to validate the robustness of the research findings comprehensively. These included the weighted median, weighted mode and MR‐Egger methods, The results of the five MR analyses involving the 3935 brain IDPs, smoking and the three psychiatric disorders can be found in Supporting Information: Tables 3–10.

### 3.3 Mediation MR Analysis

We performed mediation analyses and evaluated 3935 quantitative IDPs to assess their roles in the association between smoking and three psychiatric disorders. Our analysis identified a single mediator, the weighted‐mean *OD* (OD index) in the tract of the left uncinate fasciculus, which mediated the effect of smoking on MDD. The mediation effect was estimated as *β* = 0.106 (*p* = 0.018), accounting for 19.59% of the total effect (Table 2 and Supporting Information: Table 11). After adjusting for potential sample overlap using MRlap, the mediation effect remained statistically significant (Supporting Information: Table 15).

### 3.4 Colocalization Analysis

**FIGURE 4:**
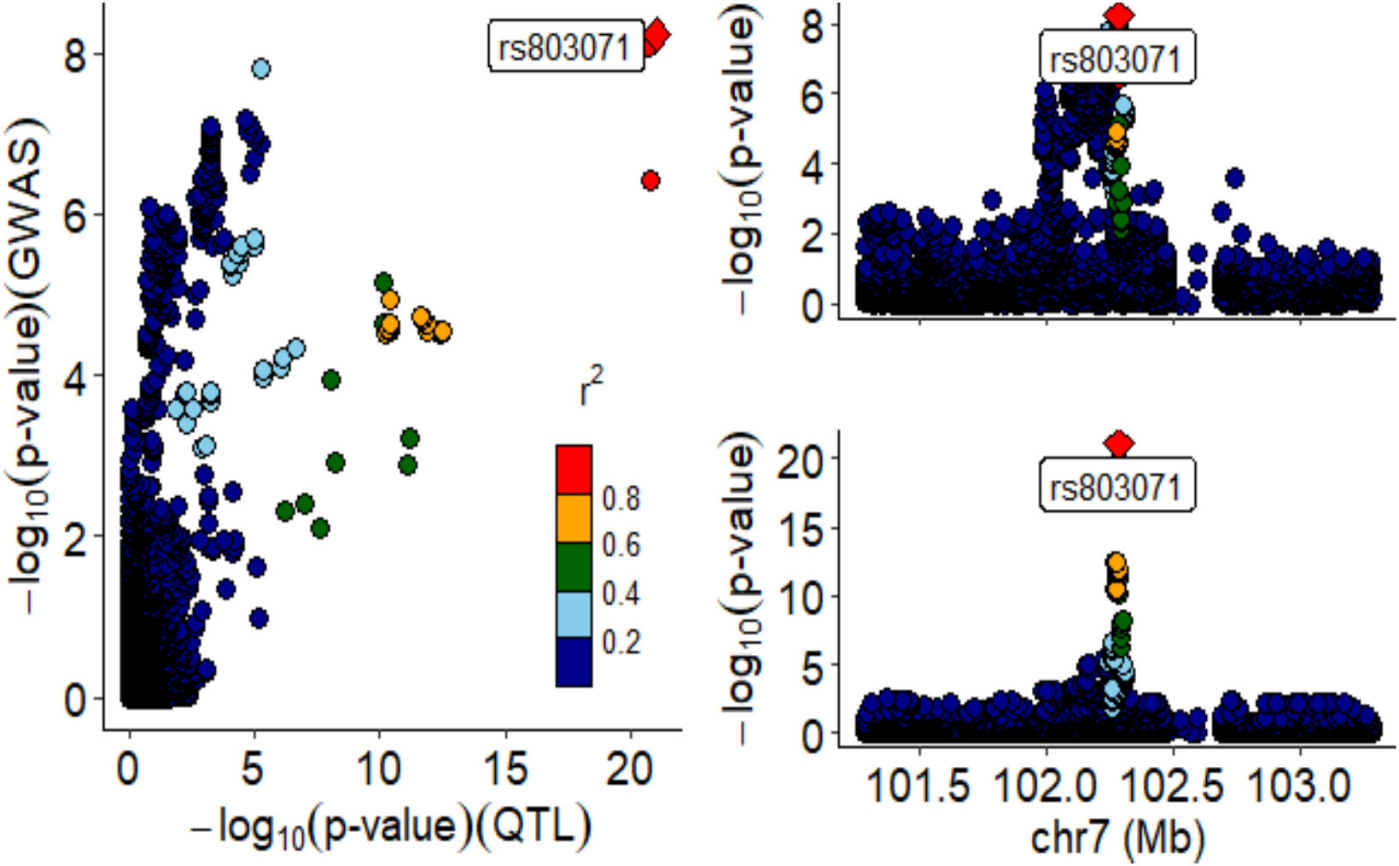
(Left) Bivariate plot of GWAS (−log₁₀[P_OD]) vs. eQTL (−log₁₀[P_eQTL]) signals in the PFC. Colours denote LD (*r* ^2^ = 0.2–0.8) relative to rs803071 (red diamond), highlighting colocalized signals. (Upper Right and lower Right) Regional association plot on Chr7: GWAS and eQTL peaks at rs803071. Locus zoom of rs803071 with *r* ^2^‐based colouring. High LD (*r* ^2^ > 0.8) supports rs803071 as the causal variant. Integrated analysis demonstrates shared genetic regulation (PP4 > 0.9) between uncinate fasciculus myelination and PFC gene expression.

**FIGURE 5:**
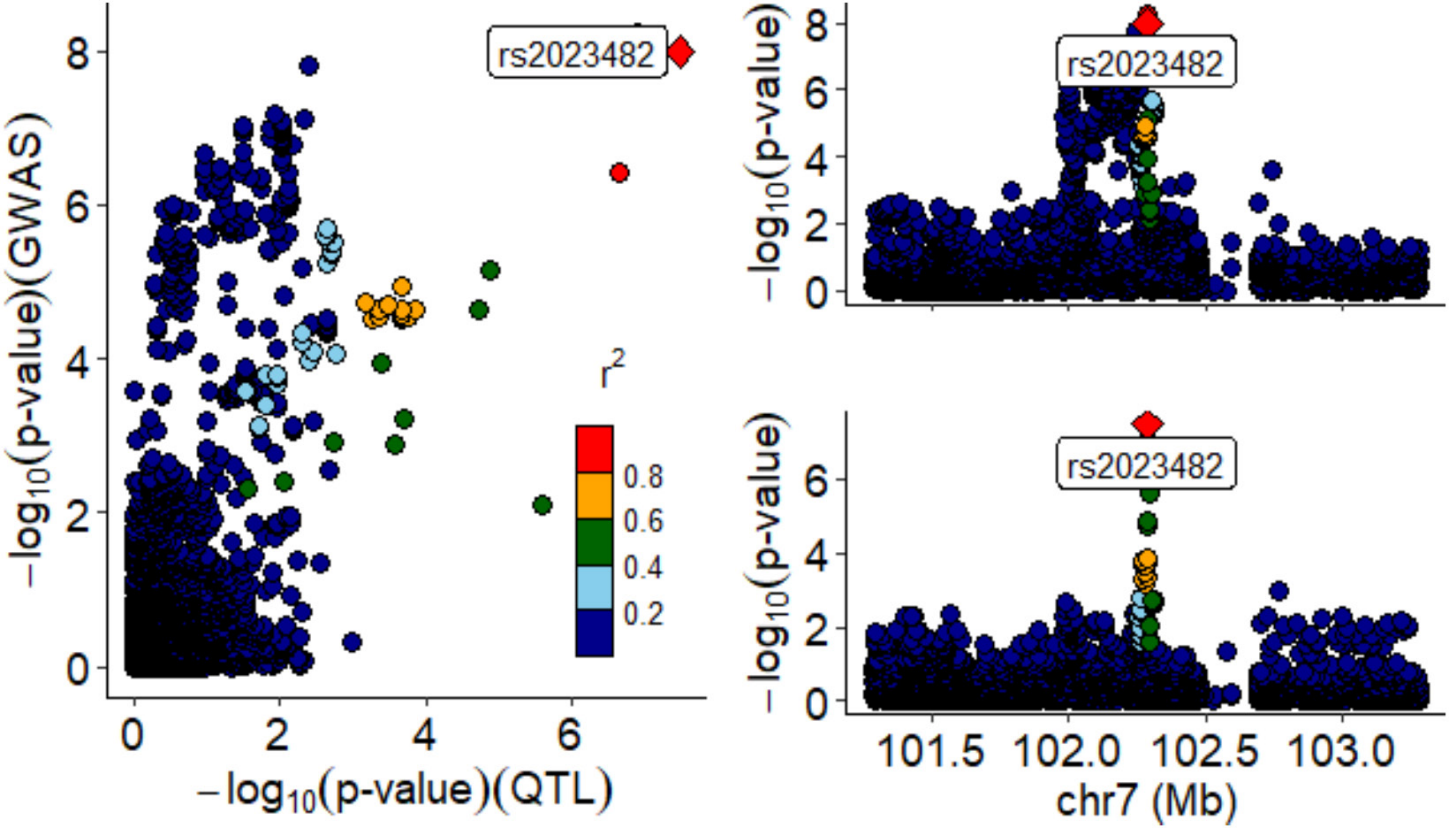
(Left) Bivariate plot of GWAS (−log₁₀[P_OD]) vs. eQTL (−log₁₀[P_eQTL]) signals in the amygdala. Colours denote LD (*r* ^2^ = 0.2–0.8) relative to rs2023482 (red diamond), highlighting colocalized signals. (Upper Right and lower Right) Regional association plot on Chr7: GWAS and eQTL peaks at rs2023482 for the weighted—mean OD in tract left uncinate fasciculus and amygdala eQTL respectively. High LD (*r* ^2^ > 0.8) supports rs2023482 as the causal variant. Integrated analysis demonstrates shared genetic regulation (PP4 > 0.9) between uncinate fasciculus myelination and amygdala gene expression.

A comprehensive colocalization analysis (coloc) and hypothesis‐driven refinement (HyPr) were conducted to explore the association between UF OD GWAS data and eQTL in the PFC and amygdala. Both analyses identified the same gene, ENSG00000160999, as having a convergent genetic effect. In the PFC, the colocalization results revealed *PPH*4 = 0.99 and HyPr = 0.98, with rs803071 identified as a functional variant candidate. Similarly, in the amygdala, the colocalization results revealed *PPH*4 = 0.97 and HyPr = 0.98, with rs2023482 identified as a functional variant candidate (Figures 4, 5, and Supporting Information: Tables 12–13).

### 3.5 Functional Mapping and Enrichment Analysis

FUMA annotation results showed that ENSG00000160999 was SH2B2, with entry 605 300 in the OMIM database, and the UniProt identifier for the encoded protein was O14492. A total of 703 protein‐coding gene overlaps (Supporting Information: Table 14) were detected between smoking, depression and weighted‐mean OD in the tract left uncinate fasciculus (UF) of GWAS, mainly located in chr17q21, chr9q33, chr3p21, chr15q21, chr16q23, and so forth (Supporting Information: Figure 3). PPI analysis showed a tightly interconnected network formed by most of the overlapping protein‐coding genes (*p* < 1 × 10^−16^) (Supporting Information: Figure 5). In tissue‐specific analysis, these overlapping genes were highly distributed in brain tissues, including the prominent basal ganglia region, PFC, cerebral hippocampus, substantia nigra and amygdala. The tissue‐specific expression pattern of each gene was visualized using an interactive heat map (Supporting Information: Figure 4).

## 4 Discussion

Our integrative analyses delineate a multilevel genetic and neurobiological framework linking smoking to psychiatric disorders, with MDD emerging as the most strongly associated phenotype. Bidirectional MR confirmed a bidirectional causal relationship between smoking and SCZ/MDD and a unidirectional causal effect of smoking on BD. Mediation analysis revealed that microstructural disorganization in the left UF mediated the effect of smoking on MDD risk.

LDSC revealed significant genetic correlations between smoking behaviour and all three psychiatric disorders, with the strongest association observed for MDD, followed by SCZ and BD. Our bidirectional MR analysis elucidated distinct causal pathways linking smoking to psychiatric disorders. Notably, smoking exhibited bidirectional causal relationships with both SCZ and MDD, whereas its association with BD was unidirectional, primarily driven by smoking‐to‐BD causality. The larger effect sizes observed for SCZ and BD compared to MDD suggest differential neurobiological interactions. We observed bidirectional causal effects between smoking and both SCZ and MDD, but only a unidirectional effect from smoking to BD. A plausible mechanistic interpretation is that nicotine more directly engages disorder‐specific nicotinic–dopaminergic circuitry in SCZ and MDD than in BD. Nicotine stimulation of α4β2 and α6 nicotinic acetylcholine receptors on ventral tegmental area (VTA) neurons enhances mesolimbic dopamine transmission [24, 25], which can transiently normalize sensory gating deficits associated with SCZ and alleviate anhedonia in MDD [26]. In contrast, in BD, the predominant neurobiological consequence of smoking appears to involve nonspecific oxidative stress and low‐grade inflammatory responses—including excessive reactive oxygen species generation, reduced glutathione levels and microglial activation. These processes are broadly neurotoxic but are less congruent with the core pathophysiology of BD, which primarily involves calcium‐channel signalling abnormalities, mitochondrial dysfunction [27] and circadian rhythm disruption [28]. Furthermore, the episodic course of BD and the antioxidant and neuroprotective effects of mood stabilizers may attenuate a consistent lifetime reverse‐causal signal detectable in Mendelian randomization analyses [29, 30]. Additionally, neurons in BD often exhibit reduced energetic efficiency and resilience, with tighter coupling between oxidative stress and intracellular Ca^2+^ dysregulation [31]. Consequently, the oxidative burden induced by smoking may precipitate mood episodes without establishing a stable dopaminergic reinforcement loop. Collectively, these features provide a neurobiological rationale for the observed pattern of causal relationships among smoking, SCZ, MDD and BD.

**FIGURE 6:**
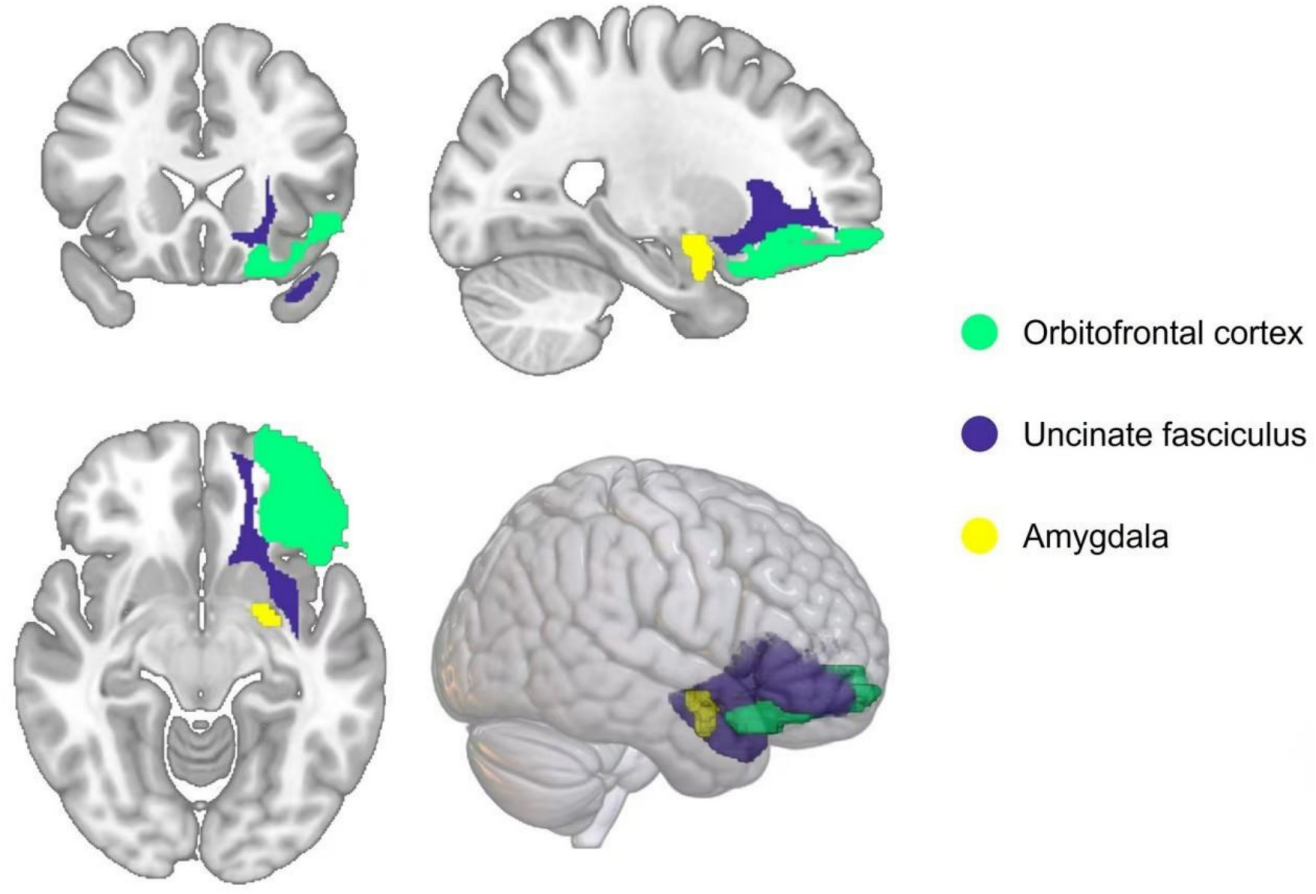
A schematic illustration of brain anatomy from axial, sagittal, coronal and 3D perspectives, showing the locations of the orbitofrontal cortex (green), the uncinate fasciculus (purple) and the amygdala (yellow). The left uncinate fasciculus originates from the orbitofrontal cortex and terminates in regions including the anterior temporal lobe.

Further mediation MR analysis quantified by the index of oriented dispersion (OD) showed that microstructural alterations in the left UF mediated the effect of smoking on MDD risk. The index of OD quantifies the angular variability of fibre orientations within a voxel and is derived from multicompartment diffusion models. Higher OD values indicate greater dispersion of fibre trajectories, such as less coherent axonal organization, increased branching or the presence of multiple crossing fibre populations. Conversely, lower OD values reflect more parallel and directionally coherent fibre bundles. In the context of the uncinate fasciculus, elevated OD may signify microstructural disorganization, including reduced axonal coherence or increased crossing complexity. Importantly, such alterations can occur even in the absence of significant changes in conventional diffusion metrics, such as fractional anisotropy (FA), and may still indicate compromised white matter integrity [32]. The UF is a white matter tract connecting the PFC and amygdala, serving as a neuroanatomical conduit through which genetic susceptibility propagates (Figure 6) [33]. Colocalization analyses included SH2B2 (APS) as a shared site for UF OD‐related variants and PFC/amygdala eQTLs, an adaptor protein that binds phosphorylated TrkB and orchestrates the dual mechanism of neuronal plasticity [34]. SH2B2 enhances TrkB‐dependent BDNF signalling and promotes synaptic resilience in the PFC‐amygdala circuit [35]. Its downregulation disrupts top‐down emotion regulation, a hallmark of MDD. Impaired SH2B2 function (e.g., rs2023482‐T) elevates UF OD values, reflecting axonal disorganization and conduction delays—a possible substrate for cognitive–emotional deficits associated with smoking [36]. Results from mediation analyses suggest that the specificity of the smoking‐MDD association compared to other disorders may stem from the unique role of SH2B2 in cortical–limbic connectivity. We hypothesize that smoking may regulate SH2B2 expression through alterations in DNA methylation, which is consistent with extensive evidence showing that tobacco exposure induces widespread epigenomic changes across the genome [37, 38]. In addition, recent smoking‐informed multiomic studies in brain tissue have demonstrated the feasibility of identifying colocalized signals between smoking‐associated GWAS loci and both methylation (meQTL) and expression (eQTL) quantitative trait loci [39]. However, our current analyses did not directly incorporate or validate SH2B2‐specific meQTLs, and publicly available brain meQTL datasets have not yet reported any clear colocalization between SH2B2 methylation sites and smoking‐associated variants. Therefore, SH2B2 hypermethylation should be considered hypothetical, pending confirmation in future tissue‐specific methylation and meQTL studies.

The convergence of genetic correlations, bidirectional causality and tract‐specific mediation underscores three key insights. First, the smoking‐MDD association is driven by pleiotropic loci (e.g., SH2B2) influencing neurodevelopmental and stress‐response pathways, indicating shared genetic architecture. Second, the UF's unique connectivity profile makes it a hotspot for smoking‐related neuropathology, bridging genetic risk with corticolimbic dysfunction and suggesting circuit‐specific vulnerability. Third, our findings offer opportunities for new therapeutic targets. Targeting the TrkB‐SH2B2 axis, in conjunction with epigenetic regulation and multimodal interventions, holds promise for disrupting the vicious cycle of smoking‐induced white matter damage and depression.

However, this study has several limitations. First, the generalizability of the findings is constrained by the predominant European ancestry in GWAS data, as ancestry‐specific differences in the microstructure of the UF are well‐documented. Second, although mediation MR analysis supports UF OD as a causal mediator of smoking effects, additional unmeasured factors, such as neuroinflammation, may account for the remaining portion of smoking's impact. Third, smoking was defined as a binary variable, which may not comprehensively capture its characteristics, such as intensity, quantity and age of smoking. Finally, the bidirectional nature of causality complicates temporal inference, necessitating longitudinal imaging‐genetic studies to determine whether UF degeneration precedes or results from depressive episodes.

## 5 Conclusion

In conclusion, our findings establish smoking as a causal risk factor for SCZ, MDD and BD, with corticolimbic white matter degeneration serving as a key mediator. Additionally, our study identifies the left UF and SH2B2 as central players in the causal effect of smoking on depression, offering a genetic framework that elucidates the cyclical interplay among polygenic risk, white matter disruption and corticolimbic dysregulation. Moreover, integrating neuroimaging techniques with genetic analyses could provide deeper insights into the underlying mechanisms. By fostering collaborations across disciplines, we can enhance our understanding of how lifestyle factors influence mental health, ultimately leading to more effective prevention and treatment options tailored to individual needs. To advance these findings toward clinical translation, future research should prioritize multiancestry cohorts, single‐cell omics and circuit‐targeted interventions, facilitating the development of precision strategies to disrupt the vicious cycle linking smoking, white matter damage and psychiatric disorders.

## Author Contributions

**Yang Chen:** writing – review and editing, writing – original draft, formal analysis and conceptualization. **Xiaoying Ma:** writing – review and editing and formal analysis. **Yubing Yin:** writing – review and editing. **Yulu Wu:** writing – review and editing. **Yiguo Tang:** writing – review and editing. **Yunqi Huang:** writing – review and editing. **Qianshu Ma:** writing – review and editing. **Siyi Liu:** writing – review and editing. **Menghan Wei:** writing – review and editing. **Mengting Zhang:** writing – review and editing. **Shiwan Tao:** writing – review and editing. **Renhao Deng:** writing – review and editing. **Min Xie:** writing – review and editing, formal analysis and conceptualization. **Mingli Li:** writing – review and editing and formal analysis. **QiangWang:** writing – review and editing, formal analysis and conceptualization.

## Conflicts of Interest

The authors declare no conflicts of interest.

## Supporting information


**Data S1:** Supporting information.

## Data Availability

The data that support the findings of this study are available from the corresponding author upon reasonable request.
