# Supplementary material for: Causal Associations Between Smoking, Brain Structural Alterations and Psychiatric Disorders: Evidence From a Mediation Analysis
Source: Addict Biol. 2025 Nov 25;30(12):e70102. doi: 10.1111/adb.70102 (PMC12646685; doi:10.1111/adb.70102)
Supplement: Supplementary file 1 — Data S1: Supporting information. [file ADB-30-e70102-s001.zip › supplementary materials/Supplementary Figure1-3.docx]

Supplementary Figure

Supplementary Figure 1 Scatter plot in the mendelian randomization analysis of smoking and psychiatric disorders.

Supplementary Figure 2 Leave-one-out sensitivity analysis in the mendelian randomization analysis of smoking and psychiatric disorders.

Supplementary Figure 3 Tissue-specific analysis of overlapping genes.

A B C


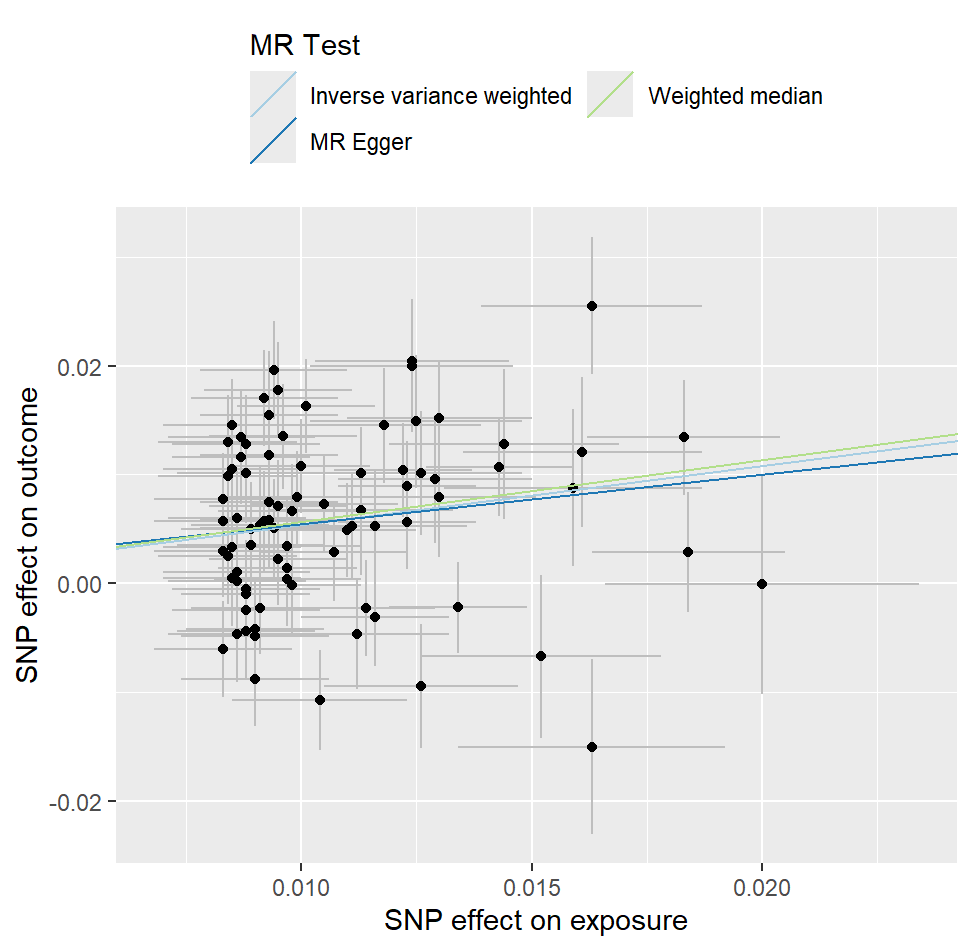

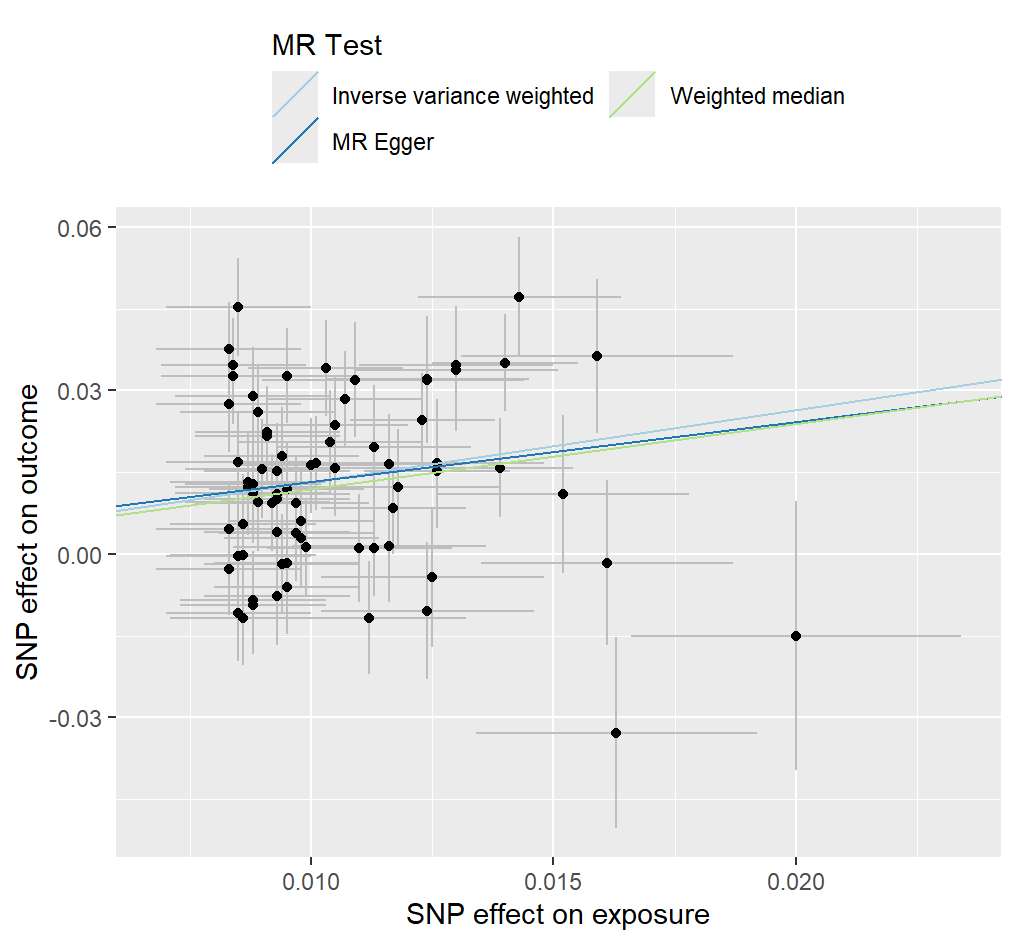

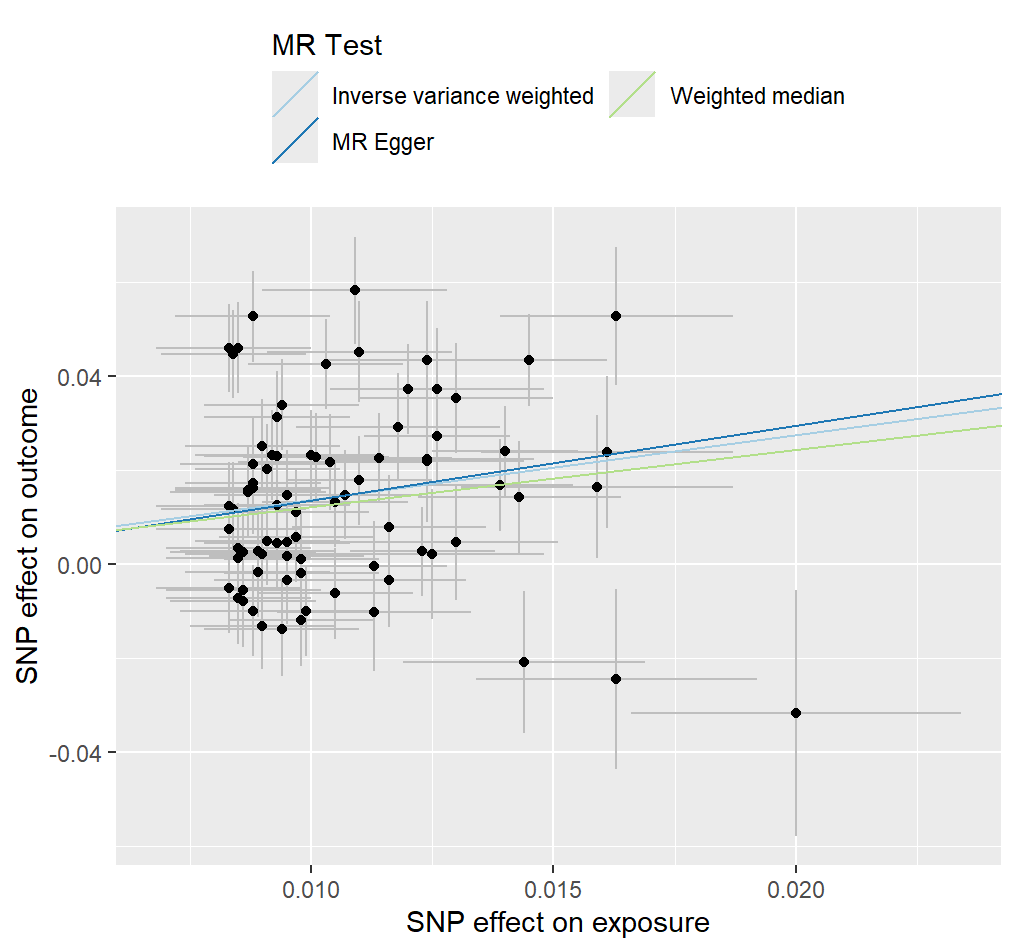


D E F


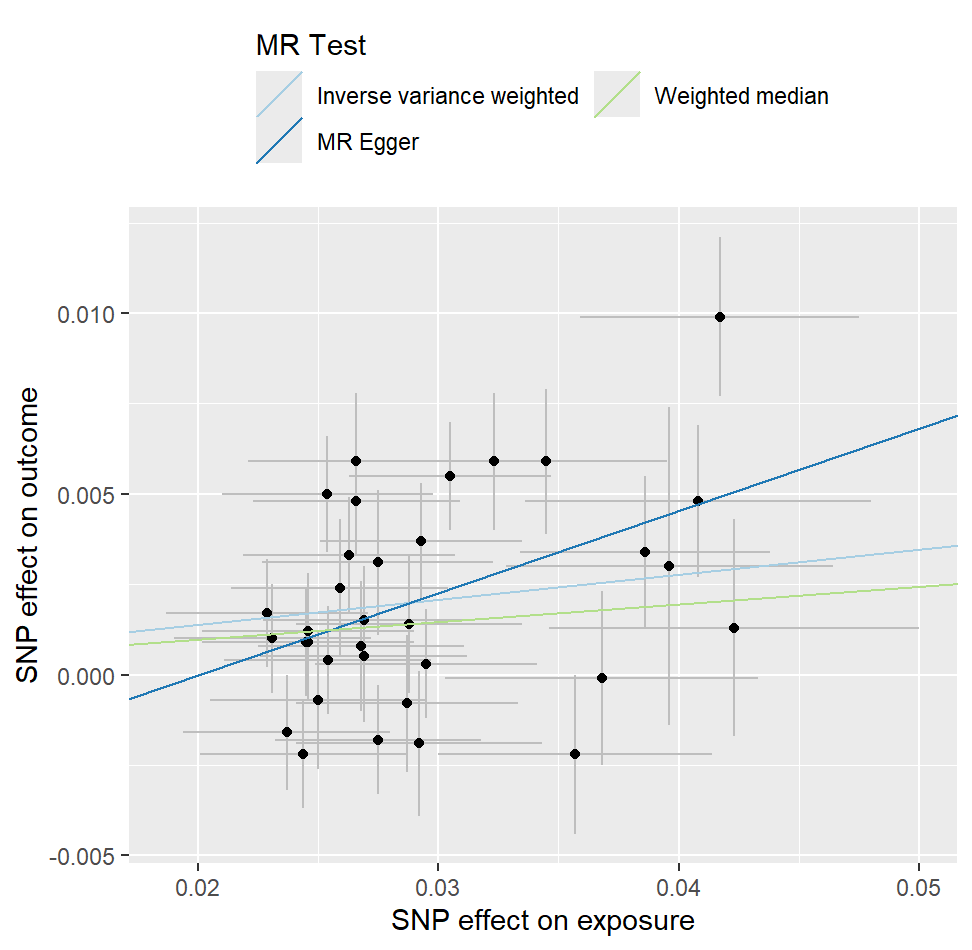

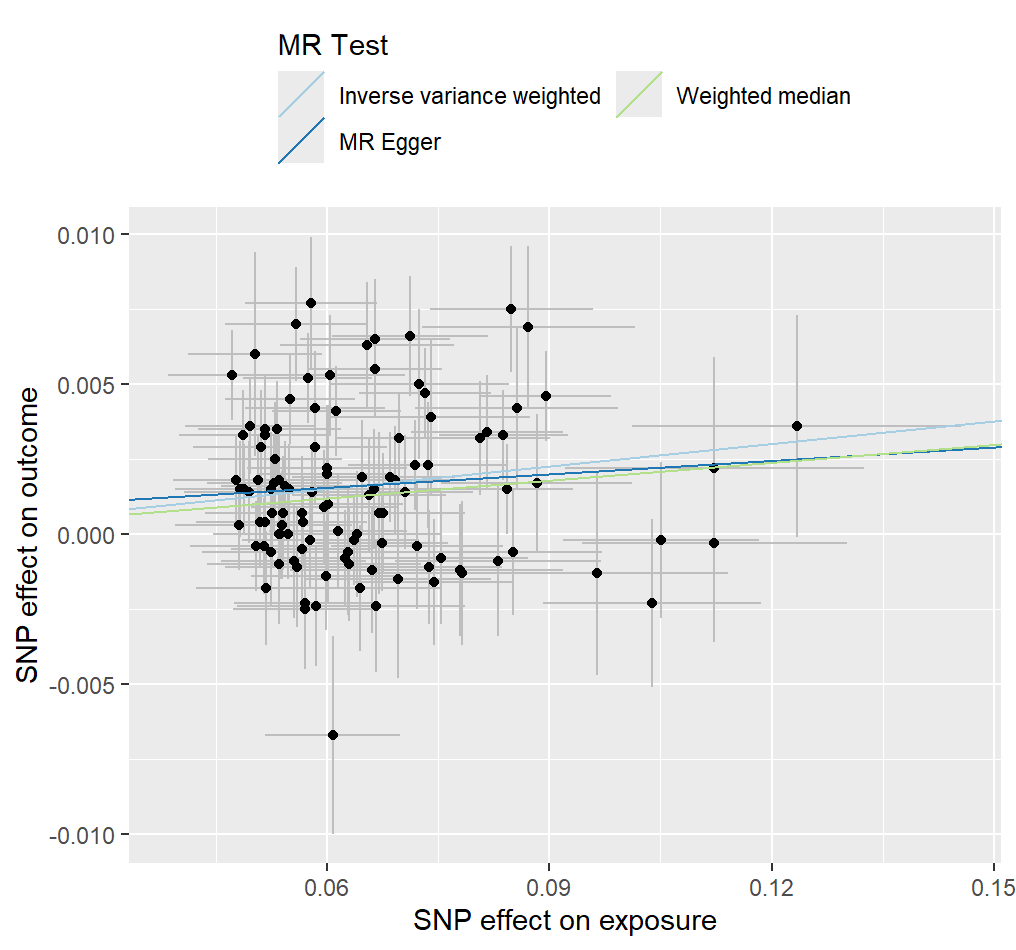

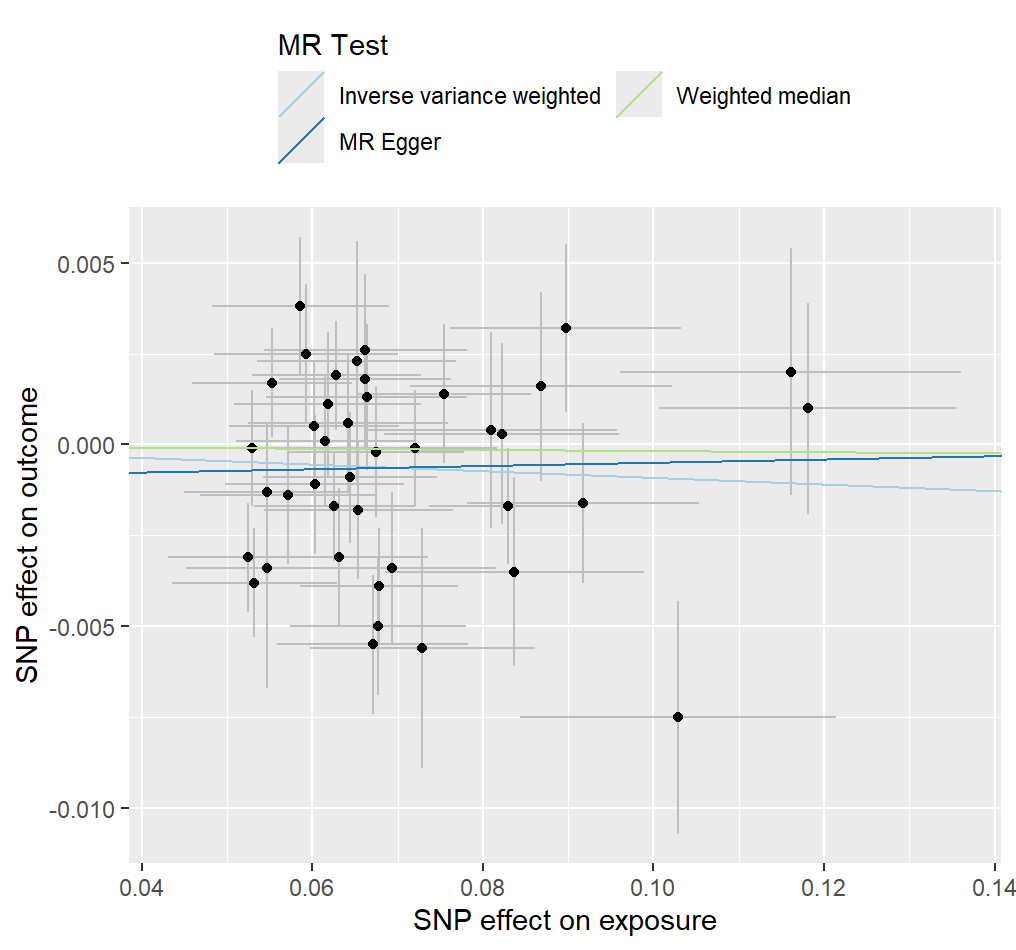


Supplementary Figure 1 Scatter plot in the mendelian randomization analysis of smoking and psychiatric disorders. (A) Causal effect of Smoking on Major depressive disorder; (B) Causal effect of Smoking on Schizophrenia; (C) Causal effect of Smoking on Bipolar disorder; (D) Causal effect of Major depressive disorder on Smoking; (E) Causal effect of Schizophrenia on Smoking; (F) Causal effect of Bipolar disorder on Smoking;

A B C


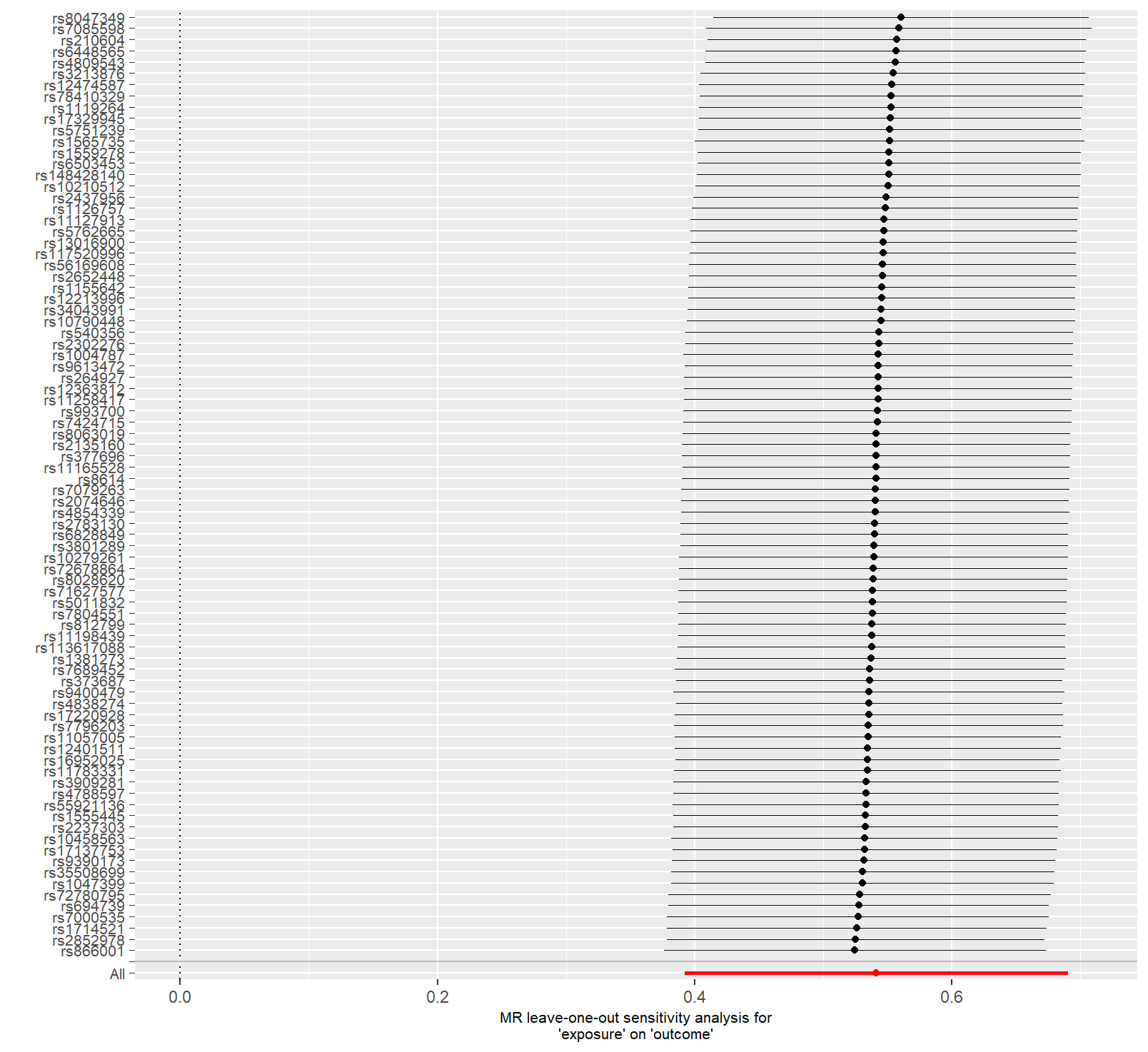

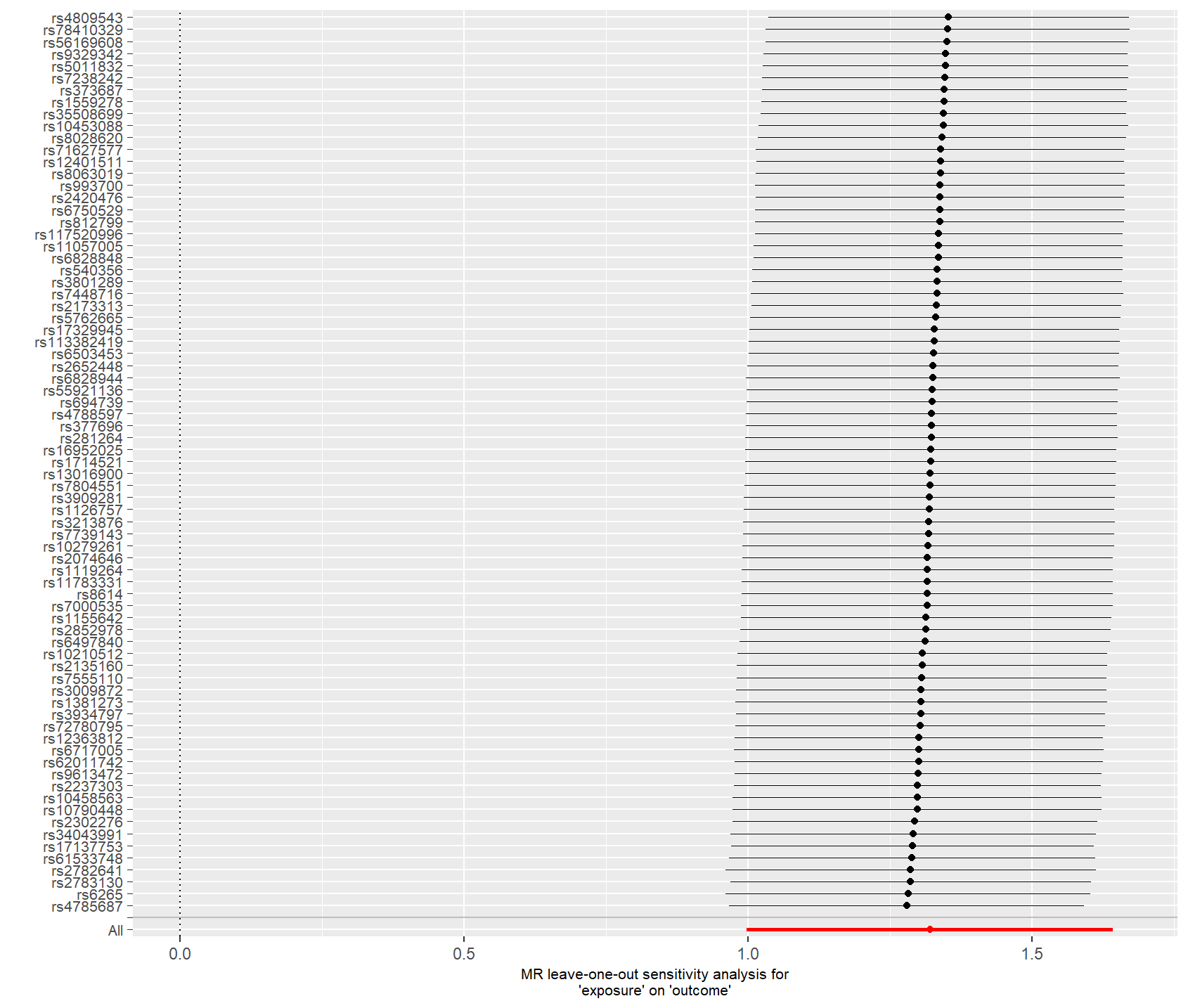

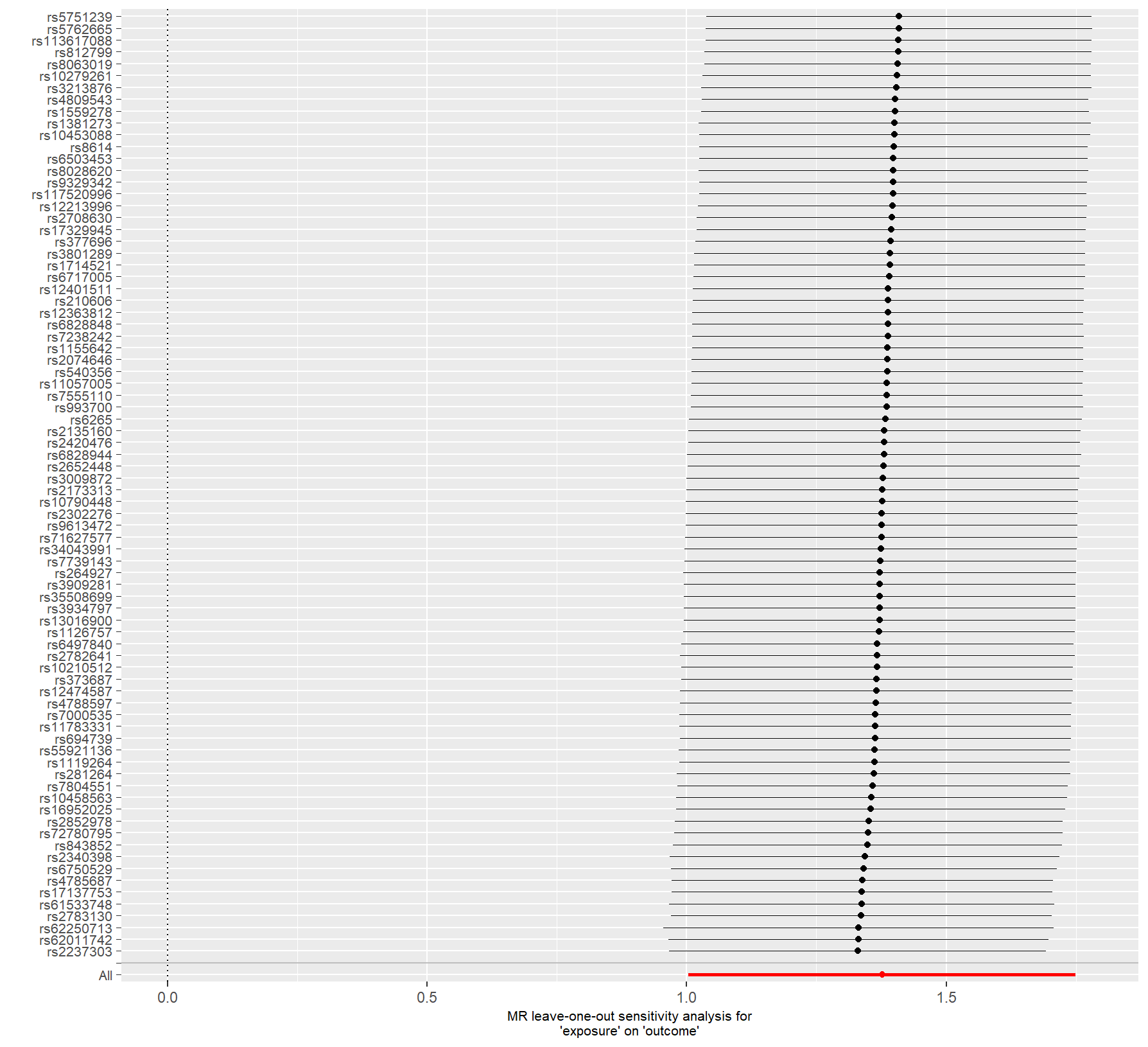


A B C


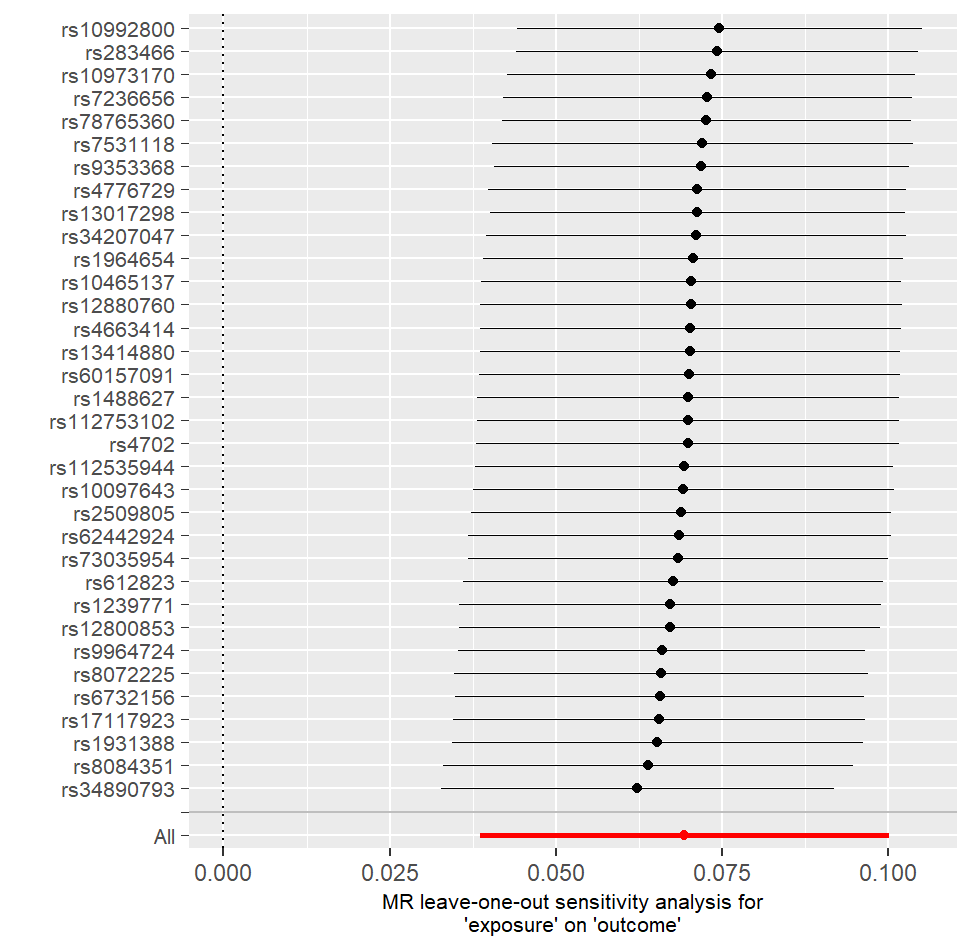

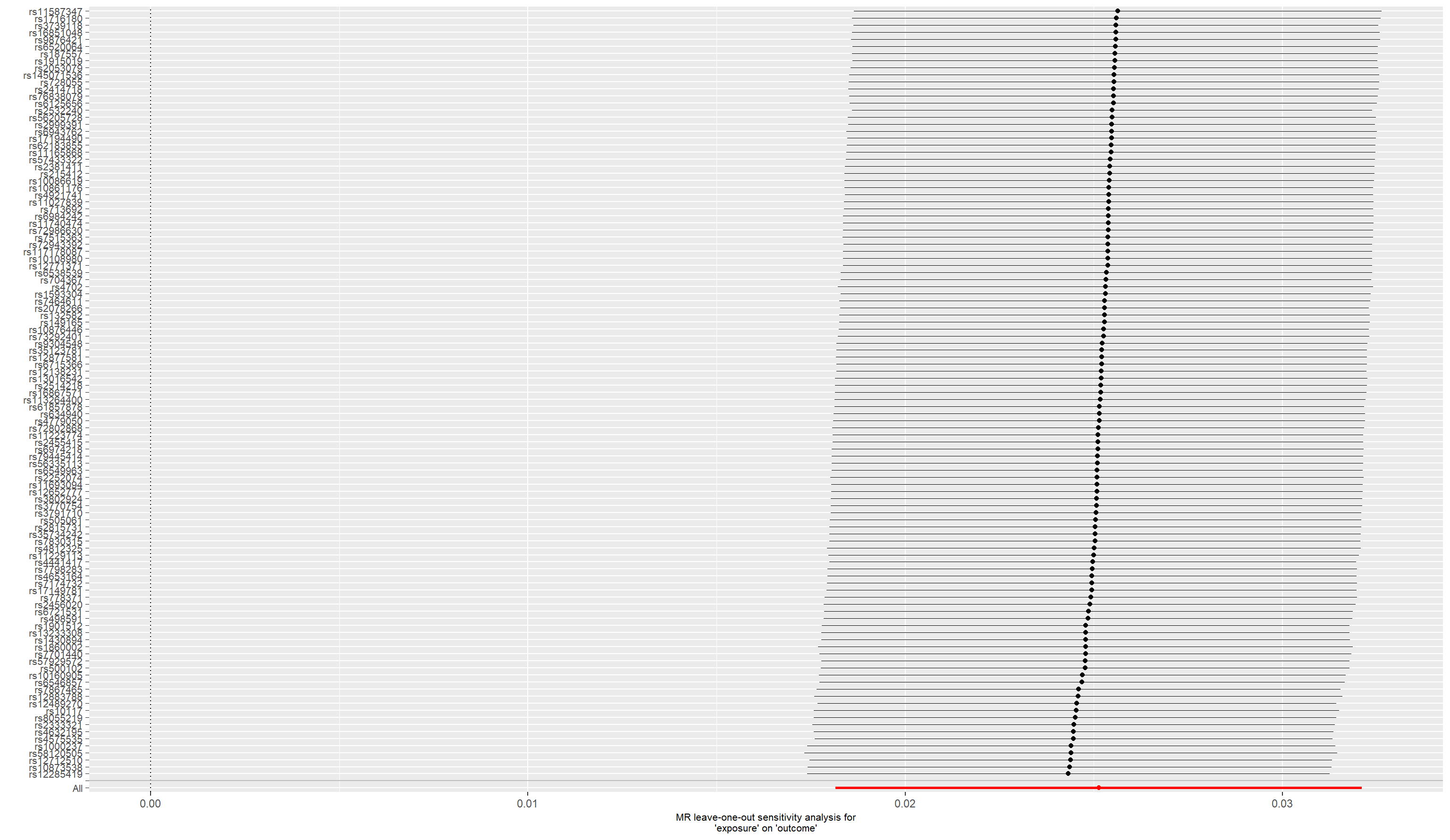

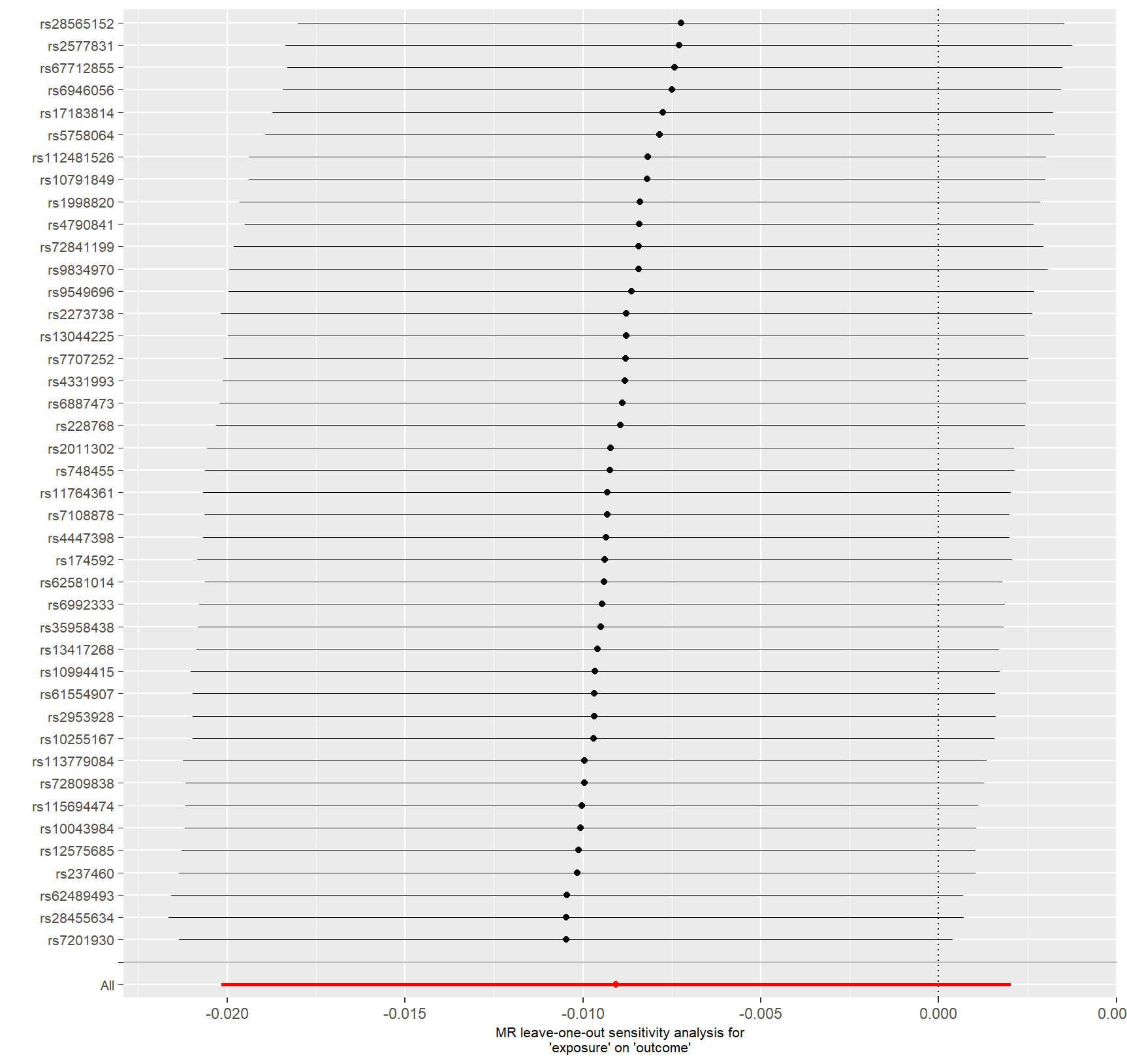


Supplementary Figure 2 Leave-one-out sensitivity analysis in the mendelian randomization analysis of smoking and psychiatric disorders. (A) Causal effect of Smoking on Major depressive disorder; (B) Causal effect of Smoking on Schizophrenia; (C) Causal effect of Smoking on Bipolar disorder; (D) Causal effect of Major depressive disorder on Smoking; (E) Causal effect of Schizophrenia on Smoking; (F) Causal effect of Bipolar disorder on Smoking;


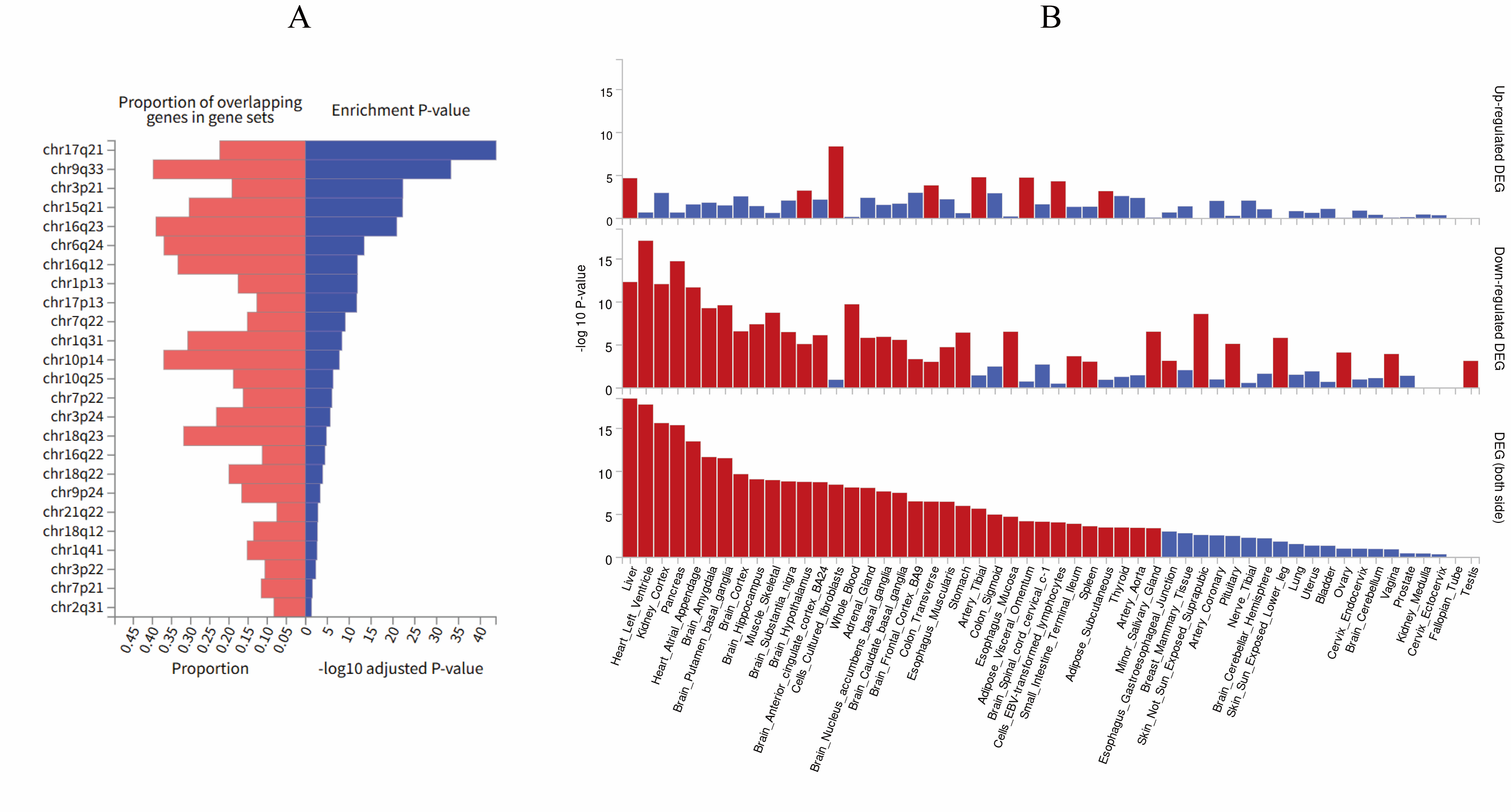


Supplementary Figure 3 Tissue-specific analysis of overlapping genes.
