# Supplementary figures and images for: Causal Associations Between Smoking, Brain Structural Alterations and Psychiatric Disorders: Evidence From a Mediation Analysis
Source: Addict Biol. 2025 Nov 25;30(12):e70102. doi: 10.1111/adb.70102 (PMC12646685; doi:10.1111/adb.70102)

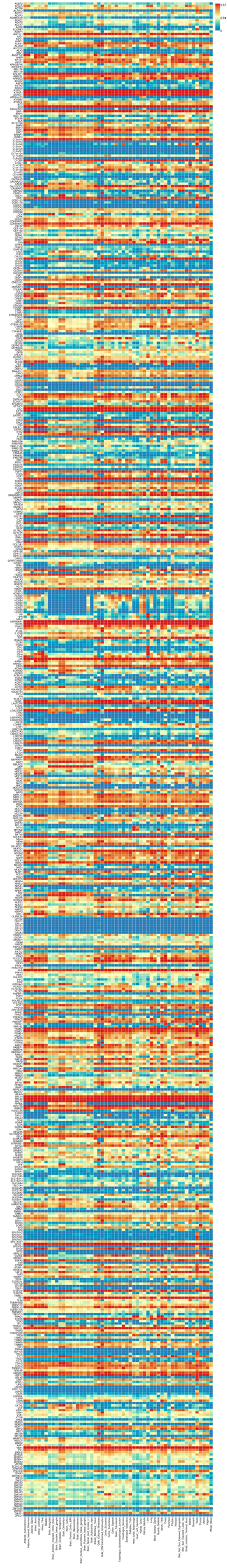

Supplement: Supplementary file 1 — Data S1: Supporting information. [file ADB-30-e70102-s001.zip › supplementary materials/Supplementary Figure_4.pdf]
